# The occurrence of symptoms of fear of COVID-19 among participants of the Polish University of the Third Age

**DOI:** 10.1192/j.eurpsy.2022.1680

**Published:** 2022-09-01

**Authors:** M. Cybulski, U. Cwalina, E. Krajewska-Kulak

**Affiliations:** 1 Medical University of Bialystok, Department Of Integrated Medical Care, Bialystok, Poland; 2 Medical University of Bialystok, Department Of Statistics And Medical Informatics, Bialystok, Poland

**Keywords:** fear, Covid-19, Older Adults, Anxiety

## Abstract

**Introduction:**

The older adults have been considered one of the groups at highest risk of SARS-CoV-2 infection and death due to COVID-19. Fear of SARS-CoV-2 infections has become widespread. It’s constantly being enhanced by the media reports and social distancing principle.

**Objectives:**

The aim of the study was to assessment the occurrence of symptoms of fear of COVID-19 among participants of the Polish University of the Third Age (UTA).

**Methods:**

The study included 296 participants of the UTA in Poland, including 258 women and 38 men. The study conducted with the use of the following validated psychometric scales: General Anxiety Disorder-7 (GAD-7), Short Health Anxiety Inventory (SHAI) and State-Trait Anxiety Inventory (STAI).

**Results:**

The mean scores in STAI and SHAI demonstrated mild symptoms indicative of anxiety disorders in the older adults. Women and men did differ significantly in terms of the scores obtained in STAI(X-1) (p=0.002) and STAI(X-2) (p=0.020). There were no statistically significant differences between respondents with higher education and those with a different level of education. The single respondents differed significantly from divorced ones in terms of STAI(X-1) (p=0.046). Moreover, widows/widowers differed significantly from divorced ones in terms of STAI(X-2) (p=0.045) and GAD-7 (p=0.032).

**Conclusions:**

The subjective experience of anxiety symptoms associated with fear of contracting COVID-19 was increased due to the ongoing pandemic, but was not significantly high in the analysed population of older people. COVID-19-related anxiety was significantly more common in lonely individuals. Women and men differed significantly in terms of perceived state anxiety and trait anxiety measured by STAI.

**Disclosure:**

No significant relationships.

